# AUD Risk, Diagnoses, and Course in a Prospective Study Across Two Generations: Implications for Prevention

**DOI:** 10.35946/arcr.v42.1.01

**Published:** 2022-01-06

**Authors:** Marc A. Schuckit

**Affiliations:** Department of Psychiatry, University of California, San Diego, La Jolla, California

**Keywords:** alcohol, genetics, sensitivity, prevention

## Abstract

This article is part of a Festschrift commemorating the 50th anniversary of the National Institute on Alcohol Abuse and Alcoholism (NIAAA). Established in 1970, first as part of the National Institute of Mental Health and later as an independent institute of the National Institutes of Health, NIAAA today is the world’s largest funding agency for alcohol research. In addition to its own intramural research program, NIAAA supports the entire spectrum of innovative basic, translational, and clinical research to advance the diagnosis, prevention, and treatment of alcohol use disorder and alcohol-related problems. To celebrate the anniversary, NIAAA hosted a 2-day symposium, “Alcohol Across the Lifespan: 50 Years of Evidence-Based Diagnosis, Prevention, and Treatment Research,” devoted to key topics within the field of alcohol research. This article is based on Dr. Schuckit’s presentation at the event. NIAAA Director George F. Koob, Ph.D., serves as editor of the Festschrift.

A large proportion of the population consume alcoholic beverages at some time in their lives. For most people, alcohol consumption is low to moderate and is not associated with harmful physiological, psychological, or social outcomes. However, for a substantial number of individuals, alcohol consumption increases over time; leads to the development of tolerance and alcohol-related life problems; and, ultimately, results in a diagnosis of alcohol use disorder (AUD). The reasons why some people develop harmful drinking behaviors and AUD are complex and still not entirely understood.

One crucial tool for identifying factors that influence alcohol consumption and its consequences are longitudinal studies that follow individuals over long periods of time, sometimes including evaluating family members over several generations. Among the most important alcohol-related longitudinal studies are the San Diego Prospective Study (SDPS), the Collaborative Study on the Genetics of Alcoholism (COGA) and the Avon Longitudinal Study of Parents and Children (ALSPAC), each of which have been supported by the National Institute on Alcohol Abuse and Alcoholism (NIAAA). This article briefly summarizes some findings from these studies, particularly the SDPS. After reviewing the contribution of genetic and environmental influences in AUD, it will introduce a low level of response (low LR) to alcohol as a risk factor for AUD. The article will then describe the 40-year SDPS in more detail, as well as its main conclusions regarding the contributions of genes and environment on the low LR and AUD, and summarizes an AUD prevention program based on the low LR.

## Genetic and Environmental Influences in AUD

The modern era of genetic studies regarding alcohol and other drug-related problems was built upon many years of observations that these problems cluster in families. Thus, children of parents with AUD have a three to four times higher risk of having AUD themselves than children of parents without AUD.^1^,^2^ However, the presence of a familial influence does not by itself demonstrate whether this familial link relates to shared genes, a shared environment, or their combination. Those distinctions were subsequently addressed in part through twin studies demonstrating that twins of people with AUD were at significantly higher risk to have AUD themselves if they were identical twins, who shared 100% of their genes, than if they were fraternal twins, who shared only 50% of their genes. An identical twin of someone with AUD has about a 60% risk of AUD compared to about a 40% risk for fraternal twins. Therefore, even in identical twins, the risk that the second twin also developed AUD was not 100%, indicating the involvement of additional factors.^3^–^5^

Additional studies examined if the enhanced risk for alcohol problems observed in children of parents with AUD remained even if the offspring had been separated from that parent early in life. In 1972, analyses of half-siblings from AUD families and control families found that adverse alcohol outcomes in offspring related more closely to presence of an AUD in a biological parent than to alcohol problems in a non-biological parent who raised the child.^6^ These data were consistent with subsequent larger and better controlled investigations of adoptees in Scandinavia.^2^,^7^ Overall, these studies supported the conclusion that genes and gene-environment interactions explained between 40% and 60% of the AUD risk.^8^–^10^

The research also indicated that genetic variants (i.e., mutations) that affect AUD risk operate in complex ways that do not fit into either dominant or recessive models of inheritance. Rather, like diabetes and hypertension, AUD can be considered a complex genetically influenced condition to which numerous genes contribute. In other words, AUD reflects the impact of multiple characteristics that do not by themselves cause the problems with alcohol but contribute to overall risk. Subsequently, research identified several genetically influenced characteristics, or intermediate phenotypes, through which genes impacting AUD risk operate.

One such intermediate phenotype is an intense alcohol-related skin flushing reaction caused by several variants of alcohol-metabolizing enzymes, which were identified in the 1970s. This phenomenon, which has been observed for centuries in people of Japanese, Chinese, or Korean descent who consume alcohol, is associated with a decreased risk for AUD but is unrelated to other types of substance use disorder (SUD).^11^ The second intermediate phenotype, which enhances risk for both AUD and other types of SUD, is the long-known association between substance-related problems and impulsive-like or externalizing behaviors.^12^,^13^ The underlying characteristics include elevated levels of sensation seeking and behavioral/physiological disinhibition. These behaviors contribute to what has been referred to as type 2 and type B subtypes of AUD that are associated with an early onset of alcohol and other drug problems and a severe clinical course.^14^ A third intermediate group of phenotypes that also is related to increased risks for both AUD and other types of SUD operates through the presence of several additional major psychiatric conditions, such as schizophrenia and bipolar disorders.^15^,^16^ Finally, this abbreviated list of genetically influenced characteristics related to the risk for AUD includes a phenotype characterized by low LR to alcohol, as described in the next section.

Each step of these studies of genetic influences for AUD also demonstrates the importance of the environment as well as gene-environment relationships. One example of data supporting the influence of environment is the finding that identical twins of individuals with AUD have only about a 60% risk for this disorder, not the 100% rate one would expect if genes explained the entire risk. Thus, it is important to study both genes and environment when looking for characteristics that might be helpful in early identification of the risk for repetitive alcohol problems or might reveal clues of ways to mitigate that risk.

## Low LR to Alcohol and Risk of Alcohol Problems

**Figure 1:**
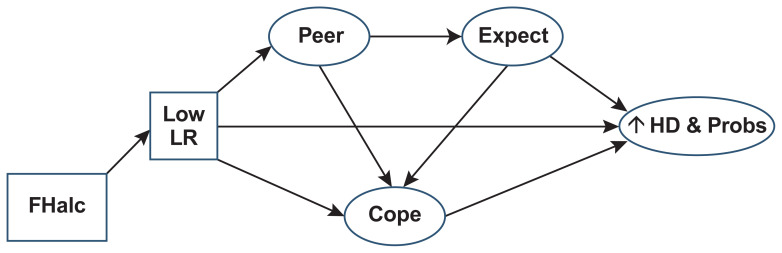
The level of response (LR) model A low LR to alcohol, which is often associated with a family history of alcohol use disorder (FHalc), increases the risk for heavy drinking and alcohol problems (HD & Probs) both directly and indirectly, through association with heavier-drinking peers (Peer), expectations that heavy drinking is desirable (Expect), and use of alcohol to cope with stress (Cope).^31^,^37^,^42^*Source:* Adapted from Schuckit et al. (2004).^19^ Reprinted with permission.

A low LR to alcohol is a genetically influenced characteristic that increases AUD risk but does not significantly impact vulnerability toward other forms of SUD or other psychiatric conditions. This low LR phenotype is most prominent at peak and falling blood alcohol concentrations (BACs).^17^,^18^ The rationale for linking a low LR with heavier drinking relates to a Social Information Processing Model which posits that individuals are likely to consume as many drinks as are needed to achieve the desired effects.^19^ According to this model, which is presented in Figure 1, young people begin drinking to achieve an effect, such as intoxication. If they need to consume more alcohol to achieve this effect—for example, because of a low LR—they will increase consumption. The resulting heavier drinking becomes associated with other outcomes, especially in individuals with a family history of AUD (FHalc), such as choosing friends who also drink heavily (Peer) or starting to expect that heavy drinking is the best way to have fun (Expect). As heavy drinking begins to increase life problems and stress, alcohol is increasingly used as a means to cope with the stress (Cope). Thus, the major impact of the low LR is on drinking quantity which then increases the risk for alcohol problems (↑HD & Probs). However, low LR has a less robust relationship with drinking frequency.^20^

The low LR is not the only response-related phenotype linked to adverse alcohol outcomes. Another phenotype is greater stimulation from alcohol, which is observed most prominently at rapidly rising BACs in some research paradigms.^21^,^22^ However, prospective work with low LR beginning in the mid-1970s forms the basis for follow-ups in the ongoing prospective study described below. Therefore, the data presented here focus on the low LR.^23^

The first documentation of the relationship between a low LR and several AUD risk factors, such as a family history of AUD, came from alcohol challenges carried out with alcohol-consuming young adults who did not have AUD but were at higher or lower AUD risk.^24^ The study compared participants at a higher risk of AUD because of a positive family history with participants at lower risk because of a negative family history who were closely matched on sex, race, percent body water, and recent drinking histories. The study found that both groups had almost identical BACs during the challenge. However, the family-history–positive group demonstrated lower intensities of response to alcohol than the family-history–negative group as measured by a range of effects, including subjective feelings of intoxication, standing steadiness (body sway), changes in hormones, and/or several electrophysiological measures.^24^–^27^

Because these alcohol challenge analyses were cost- and labor-intensive, researchers subsequently developed a less expensive and less time-consuming measure of LR that could be used in large numbers of subjects, including younger drinkers. The Self-Report of the Effects of Alcohol (SRE) questionnaire—a simple 12-item retrospective self-report—records a person’s perception of the number of standard drinks (10 to 12 grams of ethanol) required to experience up to four subjective effects (to first feel any effect, dizzy or slurred speech, unsteady gait, and unwanted falling asleep) during a typical drinking session.^28^ This instrument gathers data for three timeframes, including the approximate first five times of consuming a full drink (SRE-5), the most recent 3 months of drinking (SRE-3), and the period of heaviest drinking (SRE-H). The score for each timeframe is generated by adding the number of drinks needed for effects that the respondent has experienced and dividing that sum by the number of effects the respondent reported; this calculation yields the average number of drinks needed to achieve effects for that period. SRE values have retest reliabilities and predictive validities regarding drinking quantities and alcohol-related problems of .7 or higher.^28^,^29^ Moreover, multiple studies have documented significant positive correlations between SRE scores (i.e., needing on average higher numbers of drinks for effects or a lower LR per drink) and future heavier alcohol intake and alcohol problems.^30^–^32^

The retrospective LR measure is not identical to the alcohol challenge in which specific changes in alcohol responses are observed at rising, peak, and falling alcohol blood levels.^18^,^23^ However, laboratory measures of subjective feelings gathered at about the same time as the self-report questionnaire correlated with the SRE at >.3, and SRE ratings overlapped about 60% with alcohol-challenge results in predicting drinking quantities.^28^,^33^

## The SDPS: An Ongoing Prospective Protocol

The study comparing young adult sons of individuals who had a parent with AUD and family history controls described above progressed into the 40-year San Diego Prospective Study (SDPS), each stage of which was approved by the University of California, San Diego (UCSD), Human Research Protections Committee. The study began in 1978 with the recruitment of 453 young men (the original subjects, or probands; average age, 22 years) who were recruited through questionnaires randomly distributed to UCSD students. The participants were 18- to 25-year-old men who consumed alcohol but had never met criteria for AUD.^24^ Individuals with lifetime histories of schizophrenia, bipolar disorder, or multiple problems with alcohol or illicit drugs were also excluded.

When entering the study, probands were evaluated for low LR using oral alcohol challenges that resulted in average BACs of 60 mg/dL at 60 minutes.^24^,^34^ Probands then were followed over the next 40 years with personal interviews about every 5 years regarding changes in demography, substance use and problems, as well as major psychiatric disorders. These interviews used questions derived from the Semi-Structured Assessment for the Genetics of Alcoholism (SSAGA) instrument, which has validity, retest reliabilities, and cross-interviewer reliabilities of .7 to .8.^35^,^36^ Over the years, as probands themselves became parents, information about their children’s early development was gathered from the probands and the offspring’s mothers, and the same interviews used for the probands were also used with their children when they reached age 18 and older.

During the follow-up evaluations, probands and their children gave information on their LR to alcohol using the SRE instrument described above. Beginning with the 15-year follow-up of SDPS families, the investigators also began to record environmental and attitudinal characteristics that might partially mediate the impact of low LR on heavy drinking and alcohol problems.^31^,^37^,^38^ These mediators included:

- Perception of the maximum number of standard drinks consumed by close peers as assessed using a short version of the Important People and Activities Scale, which is scored from 0 (abstainer) to 4 (> 10 drinks) with retest reliabilities >.85 (noted in Figure 1 as Peer);^39^
- The usual effects a person expects to experience from alcohol as measured by the Social Behavior (e.g., alcohol makes parties more fun) and Increased Arousal (e.g., alcohol helps people stand up to others) subscales of the Alcohol Expectancy Questionnaires (AEQ) that are graded on a 5-point scale with an internal consistency (Cronbach’s alphas) of .72 to .92 (noted in Figure 1 as Expect);^40^
- Whether a person uses alcohol to cope with psychological problems as assessed by the Drinking to Cope scale that records how often respondents use alcohol to decrease negative emotions or boredom or to feel more confident; scores range from 1 (almost never) to 4 (almost always), and Cronbach’s alpha is .79 (noted in Figure 1 as Cope).^41^

Testing has supported the hypothetical model in Figure 1 regarding how a low LR, which occurs more frequently in individuals with a family history of AUD, increases the risk for heavy drinking and alcohol problems both directly and indirectly through these potential mediators.^31^,^37^,^42^ The findings suggested that as much as half of the impact of low LR on adverse alcohol outcomes occurs indirectly, through associating with heavier-drinking peers, expectations that getting drunk is rewarding and desirable, and using alcohol to cope with stress. These findings raised the possibility that for individuals with low LR, interventions that decrease the impact of these three mediators on heavier drinking might reduce the risk for higher maximum drinks and alcohol problems later.

## Decreasing Risk of Adverse Outcomes in People With Low LR

The findings of the SDPS served as the basis for a subsequent new study in a different population that assessed an intervention to reduce the risk of heavy drinking and alcohol problems in individuals with a low LR. To recruit participants, a questionnaire was distributed to 18-year-old students entering UCSD as freshmen to review their demography, alcohol and drug use, and related diagnoses.^43^ Potential participants also filled out the SRE to measure LR. After excluding nondrinkers and those who had been diagnosed with alcohol or drug problems, schizophrenia, bipolar disorder, or antisocial personality disorder, the researchers used a median split on the SRE to identify individuals with low and high LR, with the two groups matched on sex, ethnicity/race, and recent alcohol consumption quantities and frequencies. More than 80% of eligible students agreed to participate, and the process continued until 250 pairs of high LR and low LR respondents (500 individuals) were enrolled.

These pairs were randomly assigned to one of three conditions: One group watched four 45-minute internet-based videos that taught general ways to avoid heavy drinking and emphasized the importance of low LR (LR-based group), one group watched similar videos with information about how to limit drinking but without an emphasis on LR (state-of-the-art group), and a control group who were followed over the same 55 weeks as the first two groups but who watched no education videos. The education-group participants received $25 for viewing each of the four 45-minute lectures, one each during the first 4 weeks of the study. Students in all three groups were also paid $25 for filling out each of seven 20-minute internet-based questionnaires over the 55 weeks of the study regarding their recent drinking patterns and problems. More than 90% of participants fully participated in the protocol.

**Figure 2:**
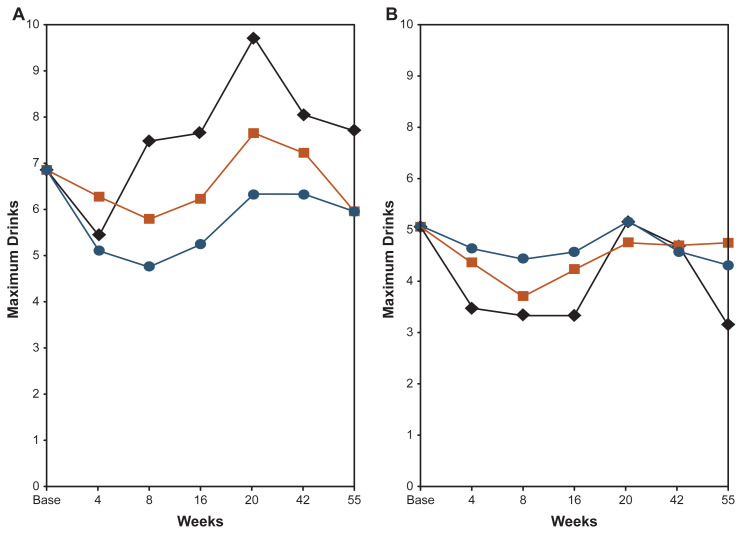
Maximum number of drinks consumed per occasion by students with low (panel A) or high (panel B) level of response (LR) to alcohol over 55 weeks in the San Diego Prevention Study Blue lines and circle symbols represent students who had watched four videos with LR-based information, orange lines and square symbols represent students who had watched four videos with general alcohol education, and black lines and diamond symbols represent control students who had watched no videos. *Source:* Adapted from Schuckit et al. (2016).^43^ Reprinted with permission.

The analyses focused mainly on the pattern of drinking quantities (i.e., usual drinks per occasion and maximum drinks per occasion) and alcohol-related problems (i.e., alcohol-related blackouts) over the 55 weeks for the three groups and the differences between the participants with low LR and high LR. Figure 2 illustrates the findings for the average maximum number of drinks; the results for usual drinks per occasion and the number of alcohol-related blackouts were similar. The left side of Figure 2, panel A, gives the average maximum drinks at each of seven timepoints over the 55 weeks for the participants with SRE scores above the median (i.e., had a lower response per drink, or a lower LR). These data are demonstrated separately for controls (in black), for the state-of-the-art group (in orange), and for the LR-based group (in blue). The right side of Figure 2, panel B, gives the results for individuals who had lower SRE scores (i.e., had higher responses per drink, or higher LRs).

The study found that among the participants with low LR, the average maximum number of drinks per occasion increased steadily over the school year, peaking during the period when the university hosted a spring celebration where heavier drinking was more common than usual. Overall, participants in the control group had the highest maximum number of drinks; the group receiving the standard-of-care intervention had significantly lower maximum numbers of drinks per occasion over the 55-week study period. The greatest reduction in maximum number of drinks, however, was found in the group who had received the LR-based intervention. Among the students who had high LR (i.e., were more sensitive to alcohol’s effects), in contrast, there were no significant changes in the maximum number of drinks over time. Moreover, no significant differences existed between the control group, the group receiving the standard-of-care intervention, and the group receiving the LR-based intervention.^43^

This study joins several others^44^,^45^ that underscore the potential importance of targeting a person’s specific preexisting vulnerability toward heavy drinking. Imparting knowledge about the genetically influenced risk factor and the mediators that amplify the impact of that risk factor can modify drinking behaviors for extended periods of time.

## Conclusions

Long-term prospective studies such as SDPS with its follow-up component provide an opportunity to evaluate problems from a unique perspective compared to other investigations.^31,43,46,47^ Such studies are challenging to carry out when funding requires renewal every 3 to 5 years, and they require great effort to ensure consistent participation over time. Thus, such investigations are costly and the number of subjects in the protocol are often limited to several hundred individuals or less, but the data that can be produced by these efforts are unique.
